# Antiepileptic Drug-Related Adverse Reactions and Factors Influencing These Reactions

**Published:** 2013

**Authors:** Parvaneh KARIMZADEH, Vahid BAKRANI

**Affiliations:** 1 Pediatric Neurology Research Center, Shahid Beheshti University of Medical Sciences, Tehran, Iran; 2 Pediatric Neurology Department, Mofid Children Hospital, Faculty of Medicin, Shahid Beheshti University of Medical Sciences, Tehran, Iran; 3 Mofid Children Hospital, Faculty of Medicin, Shahid Beheshti University of Medical Sciences, Tehran, Iran

**Keywords:** Antiepileptic drugs, Adverse reaction, Skin reaction

## Abstract

**Objective:**

According to the basic role of drug side effects in selection of an appropriate drug, patient compliance and the quality of life in epileptic patients, and forasmuch as new drugs with unknown side effect have been introduced, necessity of this research is explained. This study was conducted to evaluate the incidence and clinical characteristics of anti epileptic drug (AED) related adverse reactions in children.

**Material & Methods:**

In this descriptive study, children less than 14 years old with AED side effects referred to the Children’s Medical Center and Mofid Childeren’s Hospital (Tehran, Iran) were evaluated during 2010-2012. The informations were: sex, age, incriminating drug, type of drug side effect, incubation period, history of drug usage, and patient and family allergy history. Exclusive criterions were age more than 14 years old and reactions due to reasons other than AEDs.

**Results:**

A total of 70 patients with AED reaction were enrolled in this study. They included 26 (37%) females and 44 (63%) males. The maximum rate of incidence was seen at age less than 5 years old. All the patients had cutaneous eruptions that the most common cutaneous drug eruption was maculopapular rash. The most common culprit was phenobarbital (70%) and the least common was lamotrigine (1.4%).

**Conclusion:**

In this study, we found higher rates of drug rash in patients treated with aromatic AEDs and lower rates with non-aromatic AEDs. Various endogenous and environmental factors may influence the propensity to develop these reactions.

## Introduction

Epilepsy is a widespread chronic neurological disorder that is characterized by recurrent seizures resulting from abnormal excessive or synchronous neuronal activity in the brain and by neurobiological, cognitive, psychological, and behavioral consequences (1). According to WHO reports, there are 50 million epileptic patients in the world that 80% of them live in developing countries (2).

Two studies reported a prevalence of 1.2-1.8 for epilepsy in Iran (3,4). Drugs are the major treatment for seizure, so proper selection and use of antiepileptic drugs (AEDs) can control 60-90% of epileptic patients. Using an appropriate drug to control seizure is related to several factors, such as:

Accurate diagnosis of seizure type, Patient compliance, Drug side effects that play an important role in patient compliance.

These side effects varies from mild phenomena, such as drowsiness and mild gastrointestinal and skin symptoms to life threatening side effects, including organs failure and severe skin involvement. For instance, the mortality rate of Stevens-Johnson syndrome,which is a life threatening side effect of AEDs, can be 5-10%. Phenytoin, a common drug in seizure treatment, is the most cause of hyper-sensitivity syndrome and 10% of patients treated with phenytoin had drug eruption. Phenobarbital, another common drug, causes various skin reactions that are usually self-limited after drug discontinuation. Even in the case of newer drugs, such as lamotrigine, there are reports of life threatening side effects ranging from 1:100 in children to 1:1000 in adults. Approximately, 10% of patients used lamotrigine experienced drug eruption.

Hyper-sensitivity syndrome, which is defined by fever, rash, lymphadenopathy, and derangement of liver function test can be seen in 1/100 to 1/1000 patients treated with AEDs (5).

In different studies the prevalence of AEDs side effect varied from 10 to more than 70% (6).

As drug side effects have a basic role in the selection of an appropriate drug, patient compliance, and quality of life in epileptic patients, and as newer drugs with unknown side effects have been introduced for the treatment of seizure, necessity of this study and similar studies is explained. The objectives of the present study were to evaluate the incidence of drug reactions following ingestion of AEDs, to study both traditional and newer drugs side effects, and to investigate whether clinical factors, such as age, gender, past history, family history of allergy and using concomitant drugs influence AED-related side effects.

## Materials & Methods

This study was a descriptive study. Children less than 14 years old, with AEDs side effects referred and hospitalized in Children’s Medical Center and Mofid Childeren’s Hospital (Tehran, Iran) were evaluated during 2010-2012. All patients were visited by an Immunologist and a Pediatric Neurologist to determine the seizure and drug-induced side effects. An AED adverse reaction was defined as reaction that had no other obvious causes apart from an AED, which had caused hospitalization.

The WHO description was used for the classification of AEDs’ side effects. Required data and information (including identifying data and probable correlated risk factors) were collected by clinical evaluation and referring to the archive of hospital. These informations were as follows: sex, age, incriminating drug, type of drug side effect, incubation period, history of drug usage,, and allergy history of patients and their family. Exclusive criteria was age more than 14 years old and adverse reactions to substances other than AEDs (food, bite, non-AEDs, etc.). We compared the rate of side effects to 11 most commonly used AEDs at our center. The drugs included carbamazepine, valproic acid (VPA), phenytoin, phenobarbital, clonazepam (CZP), oxcarbazepine, lamotrigine, gabapentin (GBP), topiramate (TPM), levetiracetam (LEV), and vigabatrin. The demographic and baseline patient data and the characteristics of drug reactions were summarized using descriptive statistics. Groups were compared using Pearson’s chi-square test, Fisher’s exact test, and t-test for the categorical data. Data Analysis was carried out by SPSS 17.0.

## Results

Seventy children hospitalized due to AEDs-related cutaneous reactions were included in our study, 44 (62.9%) of the participants were males and 26 (37.1%) were females. Thirty-two (72.7%) out of 44 male cases, were less than 5 years old and 12 (27.3%) patients were more than 5 years old, whereas in 26 female cases, 16 patients (61.5%) were less than 5 years old and 10 (38.5%) were more than 5 years of age. The risk of AED- related cutaneous reactions significantly decreased with age (p=0.01).

In all patients, cutaneous reactions initiated as maculopapular rash. The most common culprit drug was phenobarbital (70%) and the least common was lamotrigin (1.4%,) (Table 1).

**Fig 1:**
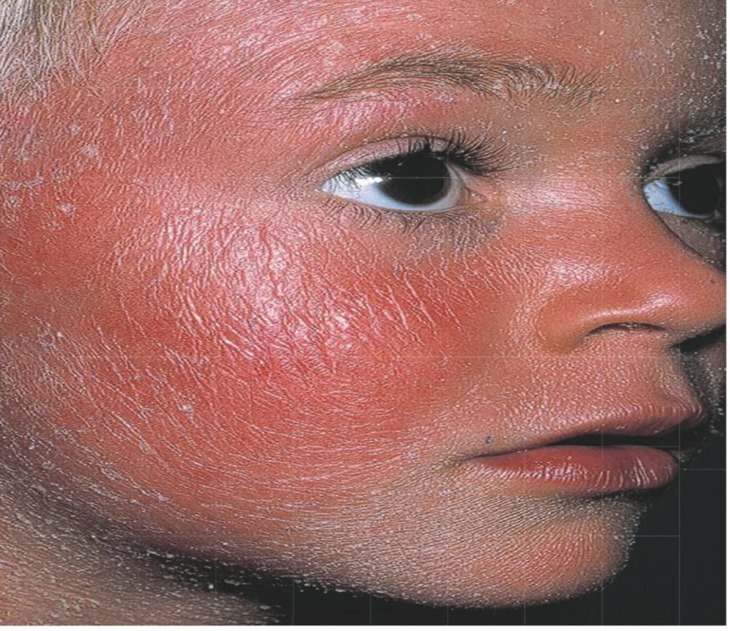
erythroderma

**Fig 2:**
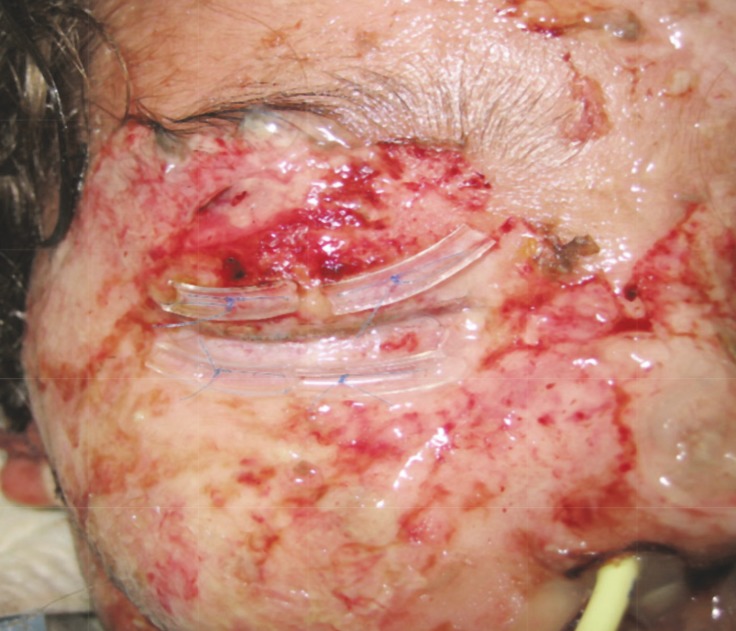
Stevens - Johnson syndrome

Eighteen patients (25.7%) had generalized erythroderma, secondary to phenobarbital in 11 patients, phenytoin in 4, carbamazepine in 2, and valproic acid in one patient. Four patients (5.7%) had macular rash, fever, leukocytosis with eosinophylia and disturbances in liver function tests, and hospitalized due to Drug Rash with Eosinophilia and Systemic Syndrome impression (DRESS) (Figure 1). In all of them the drug was Phenobarbital. Two patients (2.8%) experienced Stevens-Johnson syndrome with cutaneous eruption, at least two mucosal involvement and leukocytosis and erythrocyte sedimentation rate (ESR) incease. One with phenobarbital and another with Lamotrigine (Table 1) (Figure 2).

In 5 (7.1%) patients, symptoms initiated less than 24h after treatment, 65 patients’ symptom initiated after 24 h, and in 59 (84.2%), symptom initiation was before the first week and in 6 (8.5%) was after the first week of treatment.

**Table 1:** Drugs And Prevalence of Related Cutaneous Reaction

**Drug**	**Prevalence** **Patients(percent)**	**Maculopapular rash**	**Erythroderma**	**DRESS**	**SJS**
Phenobarbital	49 (70 %)	49 (70 %)	11 (15.8%)	4 (5.7%)	1 (1.4%)
Valproic acid	10 (14.3%)	10 (14.3%)	1 (1.4%)	0	0
Carbamazepin	6 (8.6%)	6 (8.6%)	2 (2.8%)	0	0
Phenytoin	4 (5.7%)	4 (5.7%)	4 (5.7%)	0	0
Lamotrigine	1 (1.4%)	1 (1.4%)	0	0	1 (1.4%)
Total	70 (100%)	70 (100%)	18 (25.7%)	4 (5.7%)	2 (2.8%)

15 patients (21%) were the products of consanguineous marriage and 55 (79%) were products of secound consanguineous marriage, therefore, parents relationship has no significant correlation with AEDs-related cutaneous reactions (p=0.1).There were no past history of sensitivity and atopy in 51 patients (72.9%), and 55 patients (78.5%) had no history of atopy in their first family. Thist means that patient and family history of atopy has no significant correlation with AEDs- relared cutaneous reactions. Forty-three patients (61%) experienced the side effect after the first use of AED, They had no past history of treatment with AEDs.

The contemporary use of more than one drug were seen in 24 patients, that 11 (15.7%) cases of them were treated with more than one AEDs (3 cases phenobarbital- topiramate, 3 phenobarbital-valproate, 3 valproare- lamotrigine, and 4 phenobarbital with other AEDs). Antibiotics were the more prevalent non-AEDs that were contemporarily used with AEDs.

## Discussion

Higher prevalence of cutaneous reaction in patients less than 5 years old in two genuses can be due to high prevalence of febrile convulsion and subsequently complexcFebrile convulsion (CFC). There were no past history of hypersensitivity and atopy in 51 patients (72.9%) and 55 patient had no history of atopy in their family. However, 43 patients (61%) experienced the side effect after the first use of AED without any past history of treatment with AED. This study cannot discuss the effect of allergy history on the probability of AED side effect occurance.

In all patients, cutaneous reaction initiated with maculopapular eruptions similar to other studies (7).

In Malekafzali and Najibi study on 76 patients, AEDs- related cutaneous reactions initiated with maculopapular eruptions in all cases.

Disturbances of ESR (erythrocyte sedimentation rate), CRP (C- reactive protein), and Liver function tests were seen only in six patients. In Malekafzali and Najibi study (a study about AEDs related skin reactions in all ages) leukocytosis were seen in 84% and disturbance of liver function test in 47% of cases (8). difference in systemic side effects in this study and other similar studies (laboratory data disturbances and different organ involvement) may be due to various enzymes function in drug metabolization and their metabolites that are different in various ages.

In 5 (7.1%,) patients symptoms initiated less than 24h after treatment, 65 patients’ symptom initiated after 24 h, and in 59 (84.2%) symptom initiation was before the first week and in 6 (8.5%) was after the first week of treatment, in Brandon’s study, the duration was reported from 1 day to 3 weeks (9).

The most common culprit was phenobarbital (70%) and the least common was lamotrigine (1.4%). Malekafzali and najibi reported that phenytoin is the most prevalent cause. Since our study was done on children and since phenobarbital was used in the first steps of most of the children’s seizures, the prevalence of side effect of phenobarbital is highest (8).

A comparative study is needed to determine the accumulative effect of AEDs. In other studies, the risk of side effects increases with the polytherapy (multiple AEDs) (8).

Antibiotics were the more prevalent non-AEDs in contemporary use with AEDs. In other studies, antibiotics and then AEDs are the most common cause of drug side effects (10,11).

**In conclusion**, in the present study, we found higher rates of drug reactions in patients treated with aromatic AEDs and lower rates with non-aromatic AEDs. Various endogenous and environmental factors may influence the propensity to develop these reactions. A high degree of suspicion, knowledge about risk factors, and close physician–patient contact can raise the possibility of early diagnosis and treatment. Diagnosis of severe reactions must be completely documented and reported to health authorities. The very rare occurrence of life threatening events rarely limits treatment decision-making. Future epidemiological, chemical, and genetic researches might provide methods for determining which patients are at risk, so unnecessary exposure should be prevented.
